# Role of genetic introgression during the evolution of cultivated rice (*Oryza sativa* L.)

**DOI:** 10.1186/s12862-018-1180-7

**Published:** 2018-04-23

**Authors:** Peter Civáň, Terence A. Brown

**Affiliations:** School of Earth and Environmental Sciences, Manchester Institute of Biotechnology, University of Manchester, Manchester, M1 7DN UK

**Keywords:** Allele frequency spectra, Domestication alleles, Gene flow, Introgression, *Oryza sativa*, Rice

## Abstract

**Background:**

Models for the origins of cultivated rice currently fall into two groups: ones that identify independent domestications of the *indica*, *japonica* and possibly also the *aus* types, and others that propose that the domestication phenotype was initially acquired by *japonica*, the underlying alleles then transferred by introgression to other pre-domesticated populations, giving the *indica* and *aus* varieties. Identifying the impact of past gene flow on cultivated rice genomes is therefore crucial to distinguishing between these models and understanding the domestication history of rice. To this end, we used population-scale polymorphism data to identify the progenitor gene pools of *indica*, *japonica* and *aus*. Variation shared among the cultivated groups but absent from at least one progenitor population was identified, and genomic blocks putatively transferred by gene flow among cultivated groups mapped.

**Results:**

Introgression signals were absent at the major domestication loci (*Prog1*, *Rc*, *qSH1*, *qSH3*, *Sh4*) of *indica* and *aus*, indicating that these loci were unaffected by gene flow from *japonica*. Other domestication-related loci (*Ghd7*, *LABA1*, *Kala4, LG1*) show signals of introgression from *japonica* or *indica* to *aus*. There is a strong signal for *LABA1* in *japonica*, possibly indicating introgression from *indica*. The *indica* genome is the least affected by gene flow, with just a few short regions with allelic frequencies slightly altered by introgression from *japonica*.

**Conclusion:**

Introgression has occurred during the evolution of cultivated rice, but was not responsible for transfer of the key domestication alleles between the cultivated groups. The results are therefore consistent with models in which *japonica*, *indica* and *aus* were domesticated independently, with each of these cultivated groups acquiring the domestication alleles from standing variation in wild rice, without a significant contribution from inter-group gene flow.

**Electronic supplementary material:**

The online version of this article (10.1186/s12862-018-1180-7) contains supplementary material, which is available to authorized users.

## Background

Cultivated Asian rice (*Oryza sativa* L.) is one of the oldest and most important staple crops worldwide. It is well established that *O. sativa* originated from a wild progenitor species *Oryza rufipogon* Griff., although the number of domestication events and the nature of the evolutionary processes that gave rise to the modern crop remain controversial [1–8]. Based on ecology, genetics and culinary properties, *O. sativa* can be divided into five groups – *japonica* (subdivided into tropical and temperate *japonica*), *indica*, *aus*, and aromatic rice [5, 9, 10]. Within the *japonica* subspecies, typically giving sticky rice after cooking, the temperate and tropical groups are adapted to distinct climatic conditions. *Indica* and *aus* rice are long-grained, with the latter consisting of drought-tolerant, early maturing cultivars. Aromatic rice includes cultivars with specific flavours popular in Pakistan and northern India (basmati) and Iran (sadri).

Diverse reproductive barriers have been described between *indica* and *japonica* cultivars, though the fertility of the hybrids differs from one cross to another [11, 12]. Low hybrid fertility has also been reported in crosses of either *indica* or *japonica* with *aus* [13] and aromatic rice [14]. The incomplete reproductive separation among the groups of cultivated and wild rice permits inter-population gene flow, which has been well documented in the *O. sativa*→*O. rufipogon* direction due to concerns of transgene escape from genetically modified rice [15–18].

Traditionally, studies of rice domestication have asked whether *indica* and *japonica* – the two largest groups grown in modern times – have common or independent origins. It has been shown that the evolutionary divergence of the *indica* and *japonica* genomes pre-dates their domestication by 200,000–400,000 years [19–21], which argues against the two types originating from a single domestication. Moreover, genetic structure differentiating the *indica*, *japonica* and *aus* populations has been detected by analysis of microsatellites [9], gene haplotypes [22] and genome-wide single nucleotide polymorphisms (SNPs) [3, 10]. However, the implication that the cultivated groups have independent origins is contradicted by analyses of several of the genes controlling rice domestication traits, which have revealed striking allelic uniformity across *O. sativa* [23–25]. Such observations have stimulated new hypotheses about the origin of the rice groups, proposing that some crucial domestication genes emerged in *O. sativa* only once and were subsequently transferred across other pre-domesticated populations by introgressive hybridization [3, 26, 27]. Modelling of nucleotide variation patterns have similarly led to the conclusion that either gene flow or strong artificial selection have been important demographic forces during the domestication of rice [28].

Domestication models favouring separate origins of *japonica* and *indica* followed by inter-group gene flow have also been supported by two genome-wide studies [1, 4], which showed that although the major fractions of the *indica* and *japonica* genomes are similar to distinct wild genotypes, a minor low-diversity genomic fraction groups *indica* and *japonica* together. Later analyses of rice genes apparently targeted by the domestication process have interpreted allelic uniformity as further evidence for the gene flow hypothesis [29, 30]. Most recently, the same domestication model (multiple origins plus gene flow) was expanded to *aus* by coalescent modelling of genome-wide data from several *Oryza* individuals [31]. However, none of the previous studies have demonstrated that the genomic segments supposedly involved in gene flow could not have descended vertically from the ancestral wild populations. This is either because the examined wild sample was too small to account for standing variation of the progenitors (e.g. refs. 4 and 31 analysed only two wild accessions; ref. 24 did not examine wild genotypes at all) or, more profoundly, because the ancestral wild gene pools of *indica* and *aus* have not been convincingly identified.

In a previous study, we provided evidence for independent domestications of *indica*, *japonica* and *aus* rice [5], based on examination of genomic regions that have been under selection in each of the three groups. We found that distinct sequence types had been selected in the large majority of these co-located low diversity regions (CLDGRs). This is the opposite of what would be expected if the *indica*, *japonica* and *aus* groups have a shared domestication history, as the latter should result in most CLDGRs appearing monophyletic. It has also been shown that some important domestication alleles thought to have spread across the *O. sativa* groups by introgressive hybridization are in fact widespread in *O. rufipogon* (e.g. *sh4* [32]; *rc*, *laba1* [8]) and these alleles do not always confer the domestication phenotype in wild and hybrid rice [8, 32–34]. Such observations raise the possibility that identical domestication alleles could have been selected multiple times from standing variation in independent domestication events. If this were the case, then inter-group gene flow need not be invoked as an explanation for the presence of identical domestication alleles in the different types of rice.

Understanding the extent and direction of gene flow during the evolutionary histories of *indica*, *japonica* and *aus* is therefore one of the keys to understanding the origins and domestication history of cultivated rice. We believe that a population approach with extensive sampling of wild diversity is necessary for an accurate evaluation of gene flow, and that such an analysis is lacking for rice. Prior knowledge of the progenitor gene pools is also necessary, because sharing of any genomic region among cultivated groups implies gene flow only if that genomic region is absent from at least one progenitor population. This point is particularly important in the study of crop origins, where domestication is known to have had convergent effects (e.g. all cereal crops are characterized by a lack of seed dormancy, non-shattering ear and increased kernel weight) and identical variants beneficial for cultivation and widespread in wild populations are likely to be selected multiple times. We have therefore assessed the role of gene flow by analysis of previously published genome-wide and population-scale polymorphism data [3]. We identify the progenitor gene pools of *indica*, *japonica* and *aus* by analysis of unshared ancestral variants and confirm the results using a phylogeographic approach. Subsequently, we summarize the variation shared among the cultivated groups but absent from at least one progenitor population, and then integrate our results into a comprehensive scheme of genetic ancestry that identifies genomic blocks likely to have been transferred by gene flow among the *O. sativa* groups.

## Methods

### Progenitor populations of the *O. sativa* groups

The complete genotype dataset for 1529 wild and cultivated rice accessions consisting of ~ 8 million SNPs from all 12 rice chromosomes [3] was downloaded from the Rice Haplotype Map Project database (http://202.127.18.221/RiceHap3/). Accessions with intermediate phenotypes (44) and aromatic rice (5) were discarded (Additional file 1: Table S1). The reduced dataset was split according to the group membership – 520 *indica*, 484 *japonica*, 30 *aus* and 446 wild rice accessions (using the *bash* command *cut*) – and the group SNP matrices converted into a table of allelic frequencies (using basic operations and the function COUNTIF in Libre Calc). Subsequently, only SNP positions with at least one third of non-missing data points in each analysed group were retained. A total of 705,124 variable positions passed this filter. The data filtering and analysis pipeline is schematically summarized in Additional file 2: Figure S1.

In order to identify the progenitor populations of the cultivated groups, we analysed the wild distributions of non-shared ancestral variants, by which we mean variants that are common in wild rice and one cultivated group (allelic frequency ≥ 0.05) but absent or insignificant in the remaining two cultivated groups (< 0.01). For each cultivated group, the SNP positions meeting these criteria were extracted from the SNP matrix. Subsequently, each wild accession was assessed for the presence of *japonica*-specific, *indica*-specific and *aus*-specific ancestral variants and the proportions of sites with these variants were calculated. The forty wild accessions with the highest proportions were identified for each cultivated group, yielding three non-overlapping groups of 40 wild accessions that we regard as the progenitor populations of *indica*, *japonica* and *aus*. Geographic distributions of the identified progenitors are shown on ArcGIS maps (ArcGIS software by Esri).

The robustness of the identified relationships was evaluated by a phylogenetic analysis and a principal component analysis (PCA). All format conversions and data extractions were done using *bash* command line utilities. For the phylogenetic reconstruction, a subset of the 705,124 SNP dataset was prepared by selecting 150 accessions (40 wild accessions for each progenitor population, ten *indica*, ten *japonica*, ten *aus*; the cultivated accessions with the least amount of missing data were chosen), yielding an alignment with 358,218 variable positions and 30.3% missing data points. A maximum likelihood (ML) tree was computed with RAxML [35] using the GTRCAT model, new rapid hill-climbing algorithm and 200 non-parametric bootstrap replicates. In the PCA, all variable characters from the original genome-wide SNP matrix were included, regardless of the per-site proportion of missing data, but excluding rice accessions with > 75% missing data points. The resulting SNP matrix with 701 individuals and 5,759,207 positions was analysed with smartpca [36], using the *lsqproject* option, excluding no outliers and inferring genetic distance from physical distance.

### Gene flow between domesticated groups of *O. sativa*

Gene flow among the three domesticated rice groups was examined by quantification of shared alleles and their distribution on a physical map of the rice genome (IRGSP4). First, a table of allelic frequencies in *indica*, *japonica*, *aus* and their respective progenitor populations was prepared for the 8 million SNP dataset. Subsequently, two datasets were extracted for each pair of cultivated groups; one containing variants absent in the progenitor of the first group and another with variants absent in the progenitor of the second group. In order to minimize the risk of false absence (due to small sample size of the progenitor population), only sites with at least 22 data points (out of 40) per progenitor were considered, giving a probability of 1–0.9^22^ ≅ 90% of capturing an allele that has a frequency of 0.1. Furthermore, sites with > 75% missing data in the cultivated groups were discarded (i.e. only variable positions with at least 130, 121 and eight data points for *indica*, *japonica* and *aus*, respectively, were retained). To maximize the SNP density, these filters were applied to each of the six datasets independently, yielding 965,497 alleles found in *indica* and/or *japonica* while absent in the progenitor of *japonica*; 612,951 alleles found in *indica* and/or *japonica* while absent in the progenitor of *indica*; 303,652 alleles found in *aus* and/or *japonica* while absent in the progenitor of *japonica*; 276,813 alleles found in *aus* and/or *japonica* while absent from the progenitor of *aus*; 323,665 alleles found in *indica* and/or *aus* while absent in the progenitor of *aus*; and 233,812 alleles found in *indica* and/or *aus* while absent in the progenitor of *indica*. For each of these six datasets, allele sharing was summarized as a joint allele frequency spectrum (AFS) constructed in Libre Calc (using basic operations, functions FREQUENCY and LOG10, and conditional formatting). Three additional datasets and AFSs were prepared in order to summarize sharing of alleles that are absent (≤0.01) from the entire wild superpopulation. These datasets consisted of 261,775; 162.523 and 142,381 alleles for *indica-japonica*, *indica-aus* and *japonica-aus* combinations, respectively.

The genomic distribution of the shared variants was summarized by an introgression index calculated across the entire genome in 100 kb windows with 50 kb sliding steps, using eq. 1:

$$ \overline{I}=\frac{1}{\mathrm{n}}{\sum}_i^n{p}_{iA}{p}_{iB} $$

where for every allele *i* absent in the selected progenitor, *p*_*iA*_ is the frequency of that allele in one domesticated group, *p*_*iB*_ is the frequency in the other domesticated group, and *n* is the number of alleles in a 100 kb window. We used the following assumption: if a genomic window shows an elevated proportion of alleles shared by the cultivated groups A and B and absent in the progenitor of A, then the group A obtained that region from the group B. Hence, the introgression index was calculated twice for each pair of cultivated groups (for alleles absent in each of the two progenitor populations), which summarizes gene flow in six possible directions (*indica*→*japonica; japonica→indica; indica→aus; aus→indica; japonica→aus; aus→japonica*).

## Results and discussion

### Progenitor populations of *aus*, *indica* and *japonica*

We conducted a genome-wide search for the ancestral gene pools of *indica*, *japonica* and *aus* by analysing 705,124 variable positions in the nuclear genomes of 1480 wild and cultivated rice accessions, which represent 8.8% of the genome-wide SNPs identified by [3] and roughly 0.16% of the whole genome. Following the rationale that omnipresent characters will not be informative for the resolution of potentially distinct phylogeographic origins, we focused on unshared ancestral variants, i.e. variants that are present in wild rice and one cultivated group but absent in the other two cultivated groups. This strategy does not assume independent origins of the groups a priori – under a single origin scenario there would either be no ancestral alleles specific for one cultivated group, or such alleles would always point to the same wild population. Analysis of unshared ancestral characters also enables the progenitor populations to be identified despite the confounding effects of post-domestication gene flow between the cultivated groups.

**Fig. 1:**
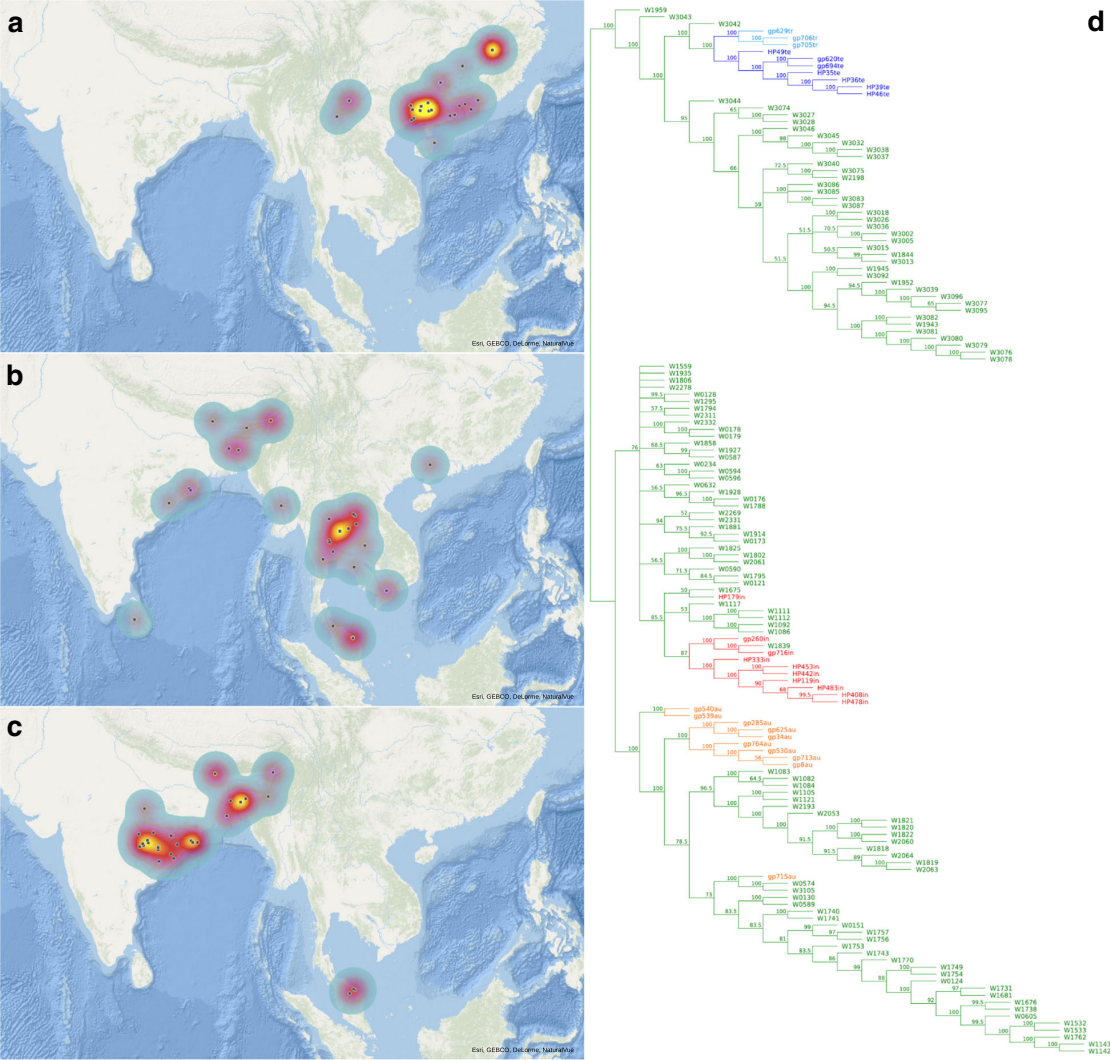
Phylogeography of wild progenitors of the *O. sativa* groups. Geographic locations of the 40 wild accessions with the highest proportions of ancestral variants specific to (**a**) *japonica*, (**b**) *indica*, and (**c**) *aus*. The heat maps take account of both the geographic density of accessions and the proportion of *aus*-, *japonica*- and *indica*-specific variants. Those accessions assigned only to country, without geographical coordinates, are placed at the mean positions for that country’s accessions: this applies to five, 14 and six accessions in (**a**), (**b**) and (**c**), respectively. (**d**) A majority-rule consensus of 200 ML trees constructed from 358,218 variable sites selected only in respect to the proportions of missing data (see Methods). The accessions are colour-coded as follows: light blue, tropical *japonica*; dark blue, temperate *japonica*; red, *indica*; orange, *aus*; green, *O. rufipogon* accessions shown on 1a–c. Bootstrap support (%) is indicated near the nodes

We identified 15,549 positions with unshared ancestral variants in *japonica*; 17,133 in *indica* and 16,162 in *aus*. We quantified these variants in wild rice and for each group we selected those 40 *O. rufipogon* accessions in which the variants are found in the highest proportions (Additional file 1: Table S1). The results (Fig. 1a-c) show that wild rice from which the *aus* group obtained its ancestral group-specific variants is distributed from the Brahmaputra valley through Bangladesh to the Odisha region in India. Wild accessions carrying the highest proportions of *japonica*-specific variants are most concentrated in the Yangtze valley and southern China. The *indica*-specific variants are not particularly concentrated in individual wild accessions; however, the samples with the highest proportions are found in Indochina and the eastern part of the Indian subcontinent.

The geography of the ancestral populations identified here is almost identical to those we previously inferred using a different approach [5], where we examined 38 CLDGRs – genomic regions with low diversity (π) co-located in *indica*, *aus* and *japonica* rice – and marked on maps those wild accessions that clustered most frequently with particular cultivated groups in neighbour-joining CLDGR-trees. In the present study, we analysed tens of thousands of ancestral, group-specific variants and we only used descriptive statistics to identify the progenitor populations. Although both analyses place the ancestral gene pools in identical geographic regions, it should be noted that the analyses may suffer from insufficient sampling. For example, a great diversity of natural wild rice populations was reported from the Gangetic plains (Uttar Pradesh and Bihar states) [37]. Unfortunately, these wild populations are under-represented in the SNP dataset [3], despite the fact that the earliest archaeological evidence of Neolithic rice exploitation on the Indian subcontinent comes from the Gangetic plains [38, 39]. The possible relationships of the wild populations from Uttar Pradesh and Bihar to cultivated *indica* and *aus* are therefore yet to be determined.

An ML tree computed from 358,218 variable positions (Fig. 1d) shows 100% bootstrap support for the monophyly of *japonica*, as well as for its association with the identified progenitor population. The *japonica* clade is further split into tropical and temperate groups with maximum support. The grouping of *aus* and its progenitor population is also fully supported, although *aus* samples do not form a monophyletic group within this clade. This could be a reflection of phylogenetic conflicts introduced by post-domestication gene flow (see the next section), or the origins of *aus* may indeed be complex. Interestingly, a previous report [40] differentiated two genetic subgroups within 250 *aus* cultivars, one of which is associated with the term ‘*boro*’, used to describe the winter growing season in Bangladesh and Assam. It is possible that *aus* and *boro* represent two cultivated groups domesticated from closely related wild gene pools. The association of *indica* and its progenitor population obtained only weak statistical support (76%) and one wild accession that we assigned to the ancestral gene pool of *indica* (W1959) is consistently resolved as basal to the (*japonica*, *japonica*-progenitor) clade (this accession was removed in the subsequent analysis of gene flow). The branching pattern within the (*indica*, *indica*-progenitor) group is largely unresolved, but all *indica* accessions are found in a clade with 85.5% bootstrap support.

**Fig. 2:**
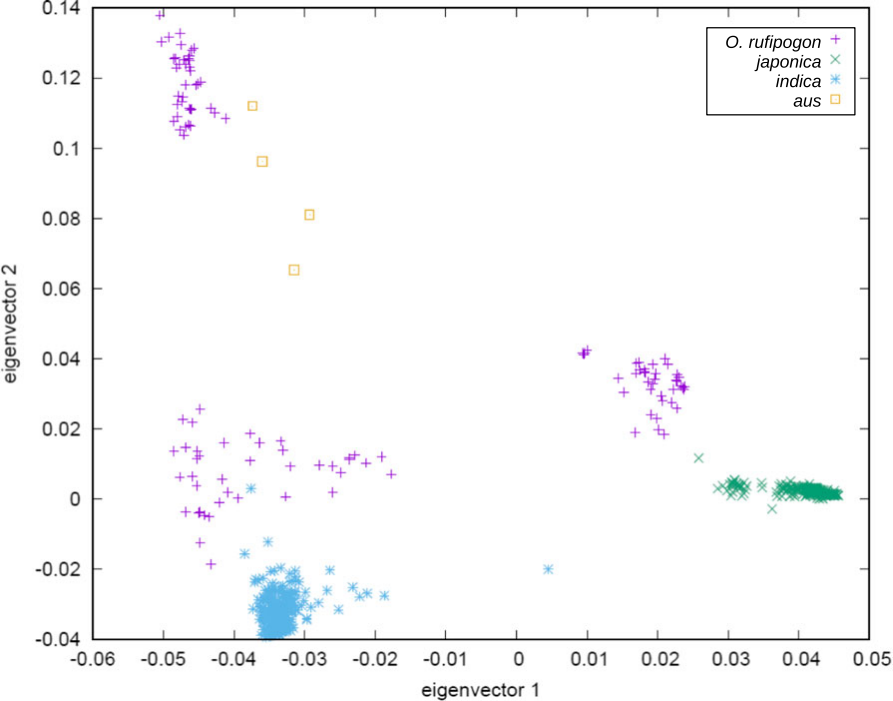
PCA of the cultivated *O. sativa* groups and the identified ancestral gene pools. A total of 5,759,207 genome-wide SNPs were transformed into 700 axes of variation. The first two axes represent 9.7% of the total variation. Rice accessions are coloured according to their domestication class

The association of the cultivated groups with their identified progenitors was further tested by PCA in which all variable positions (5,759,207) were included (Fig. 2). All cultivated groups are differentiated by the first two eigenvectors with statistical significance, except for *aus* and *indica* along the first eigenvector. Each cultivated group is closely associated with a cluster of wild accessions identified as the progenitor population in Fig. 1.

### Post-domestication gene flow among the cultivated groups

We found that 73.7% of the 705,124 polymorphic positions carry one allele simultaneously approaching fixation (allelic frequency > 0.95) in *japonica*, *indica* and *aus*. However, all of these alleles are likely to be pre-domestication variants, i.e. they are found in the wild population, usually at high frequencies (Additional file 2: Figure S2). Sharing of ancestral variants that are frequent in the wild population cannot be interpreted as evidence of common origin or inter-group gene flow, because such alleles could be obtained independently from the wild gene pool during separate domestications. For this reason, we focused on variation that is absent from at least one progenitor population, or absent from *O. rufipogon* altogether and hence a presumed post-domestication variation. Inter-group sharing of variants that are demonstrably absent from one of the groups’ progenitors can only be caused by gene flow (admixture, hybridization) or homoplasy. If the groups are in complete genetic isolation and have independent origins, sharing of such variation can only occur through homoplasy. On the other hand, if the groups shared parts of their genetic history or hybridized extensively, then the proportion of such shared variants to autapomorphies (alleles present in just one domesticated population) would increase. Quantification of the variation shared by the cultivated groups but absent from one progenitor is therefore a means of assessing the direction and magnitude of the gene flow that operated during or after the domestication of those groups.

**Fig. 3:**
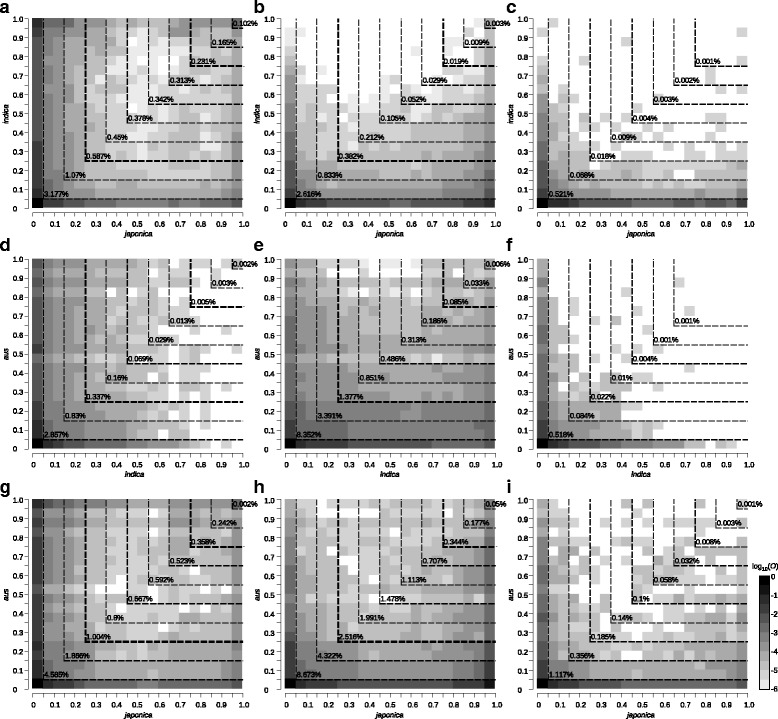
Joint distributions of allelic frequencies in *O. sativa* groups. (**a**) AFS of 965,497 *indica + japonica* alleles absent in the progenitor population of *japonica*. (**b**) AFS of 612,951 *indica + japonica* alleles absent in the progenitor population of *indica*. (**c**) AFS of 261,775 *indica + japonica* alleles absent in *O. rufipogon*. (**d**) AFS of 233,812 *indica + aus* alleles absent in the progenitor population of *indica*. (**e**) AFS of 323,665 *indica + aus* alleles absent in the progenitor population of *aus*. (**f**) AFS of 162,523 *indica + aus* alleles absent in *O. rufipogon*. (**g**) AFS of 303,652 *japonica + aus* alleles absent in the progenitor population of *japonica*. (**h**) AFS of 276,813 *japonica + aus* alleles absent in the progenitor population of *aus*. (**i**) AFS of 142,381 *japonica + aus* alleles absent in *O. rufipogon*. Axes show allelic frequency classes in respective groups. Proportions of alleles at any combination of frequency classes are indicated with logarithmic-scale shading, and also as percentages under delineated areas

A bona fide summary of genetic variation shared by two groups can be presented in the form of a multi-population AFS (joint distribution of allele frequencies across diallelic variants) [41, 42]. Typically, the alleles that are presented in an AFS are defined as ‘derived’ by reference to an outgroup. Our AFSs (Fig. 3), however, are designed to show the inter-group sharing of domestication or post-domestication variants, and therefore depict alleles that are absent in at least one progenitor population. The rationale is that if an allele is absent from the progenitor of *indica*, for example, but is shared among *indica* and *japonica*, then the implication is that the allele was transferred into *indica* after domestication, possibly from *japonica*.

We carried out AFSs of alleles present in *indica* and *japonica* but absent in the progenitor population of *indica* (Fig. 3a) or *japonica* (Fig. 3b), alleles present in *indica* and *aus* but absent in their progenitor populations (Fig. 3d,e) and the same analyses for *japonica* and *aus* (Fig. 3g,h). A common feature of all six AFSs is that > 90% of the alleles absent in one progenitor are either unshared or shared below 0.05 frequency by the two cultivated groups. This indicates that the major parts of the *indica*, *aus* and *japonica* genomes have been unaffected by inter-group gene flow. Very few alleles (0.002–0.102%) absent in one of the progenitors are fixed in both cultivated groups, further indicating that only a small fraction of alleles transferred by gene flow was globally subjected to selection. The *aus* group appears to be the most affected by the gene flow, both from *indica* (Fig. 3e) and *japonica* (Fig. 3h), sharing with these two groups 8.352% and 8.673% of alleles absent in the *aus* progenitor, respectively. Nonetheless, only a minute fraction of these alleles reach fixation. On the other hand, the genome of *indica* appears to be the least affected by gene flow, sharing only 2.616% and 2.857% of alleles absent in its progenitor with *japonica* (Fig. 3b) and *aus* (Fig. 3d), respectively.

The proportions presented on the AFSs are good indicators of the inter-group gene flow if the following three conditions are met: (i) the progenitor populations identified here are a good representation of the actual progenitors at the beginnings of agriculture; (ii) the gene flow operated in the direction crop→crop and not in the direction wild→crop; and (iii) the absence of alleles in the progenitors is correctly determined and does not result from low sample size, missing data or recent allele extinction. The first condition cannot be established, since an exact reconstruction of past demographic processes is not possible. Although we demonstrated significant association of the cultivated groups with their identified progenitors (Figs. 1 and 2), it is possible that the original gene pools were not sampled here (see above), or their diversity was different.

**Fig. 4:**
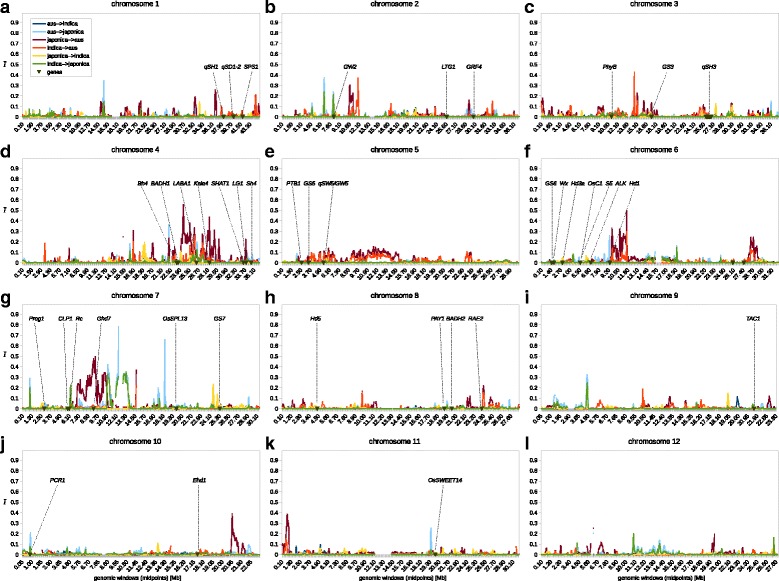
Genomic map of regions introgressed among *O. sativa* groups. Introgression index (*y*-axis) calculated in 100 kb genomic windows (*x*-axis) across all 12 rice chromosomes (**a**-**l**). Positions of genes and quantitative trait loci known to be relevant for cultivation are indicated with dark green arrowheads

If it was wider, the proportions of shared alleles are overestimated; if it was narrower, the proportions are underestimated. To tackle the possible over-estimation together with the second issue (ii), we constructed AFSs for alleles absent in the entire wild superpopulation (Fig. 4c,f,i). As these AFSs are limited to post-domestication variation (variation that arose in the cultivated groups at any time since the domestication) and exclude wild→crop gene flow, the proportions of shared alleles decrease substantially. Very low levels of post-domestication gene flow between the three cultivated groups are implied, with the strongest signal recorded between *japonica* and *aus* (Fig. 3h).

The third issue (iii) can be examined by looking at the genomic distribution of the shared alleles. Shared alleles with erroneously established absence in the progenitor are expected to be randomly distributed across the genome. Consequently, clustering of the shared alleles in a particular genomic region points at introgression from a different population. For this purpose, we performed a genomic scan of the introgression signal, using the product of allelic frequencies for a pair of domesticated groups calculated for all sites where the allele is undetected in one of the progenitors (eq. 1; Fig. 4). This introgression index is averaged for 100 kb windows with 50 kb sliding steps, and simultaneously measures allelic similarity in the cultivated pair and their dissimilarity from the selected progenitor. The index value can vary from zero – indicating no introgression (when no shared variants absent in the progenitor are found), to one – indicating total introgression (when all variants absent in the progenitor are fixed in both cultivated groups).

Interestingly, regions containing alleles responsible for the erect growth (*Prog1* [25]; Fig. 4g), white pericarp (*Rc* [24]; Fig. 4g) and non-shattering ear (*qSH1*, *qSH3, Sh4* [23, 33, 43]; Fig. 4a,c,d, respectively) show nil signal of introgression in *indica* and *aus*, which implies that the two groups obtained these genomic regions directly from their progenitors. In the case of *japonica*, weak signal of introgression from *indica* was detected in the region containing the *Rc* gene. However, it is not clear whether the *Rc* gene itself was involved in this putative introgression block, since the signal is detected 23–167 kb downstream of the *Rc* coding sequence. If the *rc* allele was indeed introgressed into *japonica* from *indica*, the direction of that introgression is the opposite of the one concluded previously [24]. However, the previous conclusion was based on an assumption that the recessive *rc* allele had evolved from red-grained varieties under cultivation, which we find to be unsubstantiated [8].

Introgression signals in the same direction and with similar magnitude were detected 17–125 kb upstream of the *GW2* gene (Fig. 4b), and unambiguously spanning the *LABA1* gene (1.8 Mb around the coding sequence; Fig. 4d). The *GW2* gene has a modest effect on grain size and shows signs of purifying selection [44]. Since distinct alleles of *GW2* are prevalent in *indica* and *japonica* [44], introgression of the coding sequence into *japonica* seems unlikely. The recessive *LABA1* allele is responsible for the short and barbless awns in cultivated rice [29]. Since the *LABA1* region in the progenitor of *japonica* is rather dissimilar from the one in *japonica*, it appears that the locus was introgressed from *indica*. This direction of introgression is again the opposite of the one suggested previously [29], but we note it is unclear how the direction was established in that study. Interestingly, the *laba1* allele is not fixed in temperate *japonica* which often retains barbed awns [8, 29], indicating that the introgression into *japonica* was not global. Additionally, a mild signal of introgression into *japonica* was detected 45–79 kb upstream of the *Hd1* gene (Fig. 4f). Variations in the *Hd1* gene on chromosome 6 cause changes in photoperiod sensitivity, a trait that can be crucial for cultivation in some areas [45]. Our findings suggest that this locus may have been introgressed into some *japonica* varieties from *aus*. The genome of *japonica* shows additional introgression signals (e.g. on chromosome 7, Fig. 4g), however, these are distant from genes known to be involved in domestication.

Multiple genomic regions were introgressed into the genome of *aus*, either from *japonica* or *indica*. The strongest signal was detected in the direction *japonica*→*aus* involving a 3.5 Mb region on chromosome 7 surrounding the *Ghd7* locus (Fig. 4g). The *Ghd7* gene has a pleiotropic effect on plant height, heading date and yield, and its haplotype structure suggests independent selection during the domestication of *indica* and *japonica* [46]. Here we conclude that this locus was subsequently transferred from *japonica* to *aus*. Other signals of introgression into *aus* were detected on chromosome 4, involving the genes *Kala4* and *LG1* (Fig. 4d). The *Kala4* gene regulates expression of several genes responsible for pigment production [47] and the *LG1* gene is responsible for the compact panicle architecture in cultivated rice [48]. Our results suggest that both genes were introgressed into *aus* from *japonica*. Additional strong signals of introgression into *aus* from either *indica* or *japonica* were also detected on chromosomes 1, 2, 4, 6, 10 and 11 however, these regions do not contain known domestication alleles.

Only two weak introgression peaks were detected in the genome of *indica* – short regions on chromosomes 4 and 7 that appear to originate from *japonica* (Fig. 4d,g). To the best of our knowledge, these regions do not contain known domestication-related genes.

Due to the amount of missing data in the original SNP matrix [3], we were only able to examine a fraction of the variable sites observed in wild and cultivated rice. Nonetheless, we analysed data for 96.5% of the genomic windows, and the introgression index was typically calculated from tens to hundreds of sites per window (Additional file 2: Figure S3). Hence, our analyses capture almost the entire genome and the introgression index is robust across most of its length.

Recently, the ABBA-BABA test and coalescent modelling on several *Oryza* individuals with de novo assembled genomes have been used to infer ancestral population sizes, divergence times and gene flow rates [31]. The authors estimated that *indica* obtained 17% of its genome from *japonica*, and *aus* obtained 15% and 11% of its genome from *japonica* and *indica*, respectively. Although our analyses do not yield exact quantification of the genomic fractions subjected to introgression, the conclusions of Choi et al. [31] do not appear consistent with our results, particularly in case of *indica*. We believe that there are several potential sources of bias in the approach used by Choi et al. [31]. First, since the authors did not analyse populations, the individuals used for the coalescent modelling may not be representative of the gene pool compositions in the *japonica*, *indica* and *aus* groups. Particularly, the *indica* cultivar IR64 used for their coalescent modelling has a complex pedigree with several *japonica* parents [49] and the second *indica* cultivar used by Choi et al. [31], 93–11, is also known to have a *japonica* cultivar in its pedigree [50]. Such recent hybrid history could seriously affect the gene flow estimate, which will therefore not be representative of the *indica* group as whole. Since modern cultivars are genetically altered by breeding efforts, traditional landraces are the preferred material for crop domestication studies. The domesticated dataset utilized in our analyses [3], consists almost entirely of landraces. Moreover, both coalescent reconstruction and the ABBA-BABA test are models of neutral evolution that cannot account for selection. Accordingly, the authors [31] have chosen and analysed genomic regions void of signs of selection. On one hand, this is the correct application of the methods, but on the other hand it means that coalescent modelling and the ABBA-BABA test cannot inform us about the fraction of the rice genome that was under selection during domestication, and therefore cannot answer the questions about the origin of domestication alleles.

## Conclusions

Considerations of gene flow among *O. sativa* groups need to recognize the existence of the partial reproductive barrier between *indica* and *japonica* [11, 12], their different ecological requirements, and the unusually high pairwise F_ST_ values (Additional file 1: Table S2), all of which argue against there having been high levels of genetic interaction. Nonetheless, gene flow between *indica* and *japonica* is a popular concept, because it offers a simple explanation for the conundrum of why some genetic loci appear identical in these two groups despite their generally distinct genetic architectures. Several models of rice domestication have hypothesized that gene flow occurred in the early stages of rice cultivation [26, 27] and was crucial for the emergence of the non-*japonica* groups [3, 31]. These hypotheses assume that some of the domestication alleles were acquired from local standing variation or emerged under cultivation (through unique mutations), were targeted by artificial selection and – because of their superior phenotypes – were introgressed into other proto-domesticated populations where they became fixed and helped to establish the domestication phenotype.

One genetic expectation of such a model is the existence of alleles shared by the cultivated groups at high frequencies while absent from the progenitor of the recipient group. Here we tested this expectation and examined clustering of such alleles, which we interpret as a signal of introgression (Fig. 4). We did not detect any introgression signal at the major domestication loci *Prog1*, *Rc*, *qSH1*, *qSH3* and *Sh4* in *indica* and *aus*, which implies that gene flow from *japonica* was not involved in the establishment of crucial domestication characteristics in *aus* and *indica*. Other domestication-related loci in the genome of *aus*, namely *Ghd7*, *Kala4* and *LG1*, show signals of introgression from *japonica*. The genome of *japonica* also shows signals of introgression, some of which are associated with domestication-related loci (unambiguously with *LABA1*, tentatively with *GW2*, *Hd1* and *Rc*). Interestingly, the *indica* genome appears to be the least affected by gene flow, with a couple of introgression peaks on chromosomes 4 and 7, although distant from known domestication-related genes. Another important observation is that among 261,775 post-domestication variants occurring in *indica* and/or *japonica* (Fig. 3c) there is not a single allele simultaneously fixed (> 0.95) in both groups, while only seven alleles are shared at frequencies > 0.5. All of these observations, together with our genome-wide phylogenetic and population diversity analysis (Fig. 1, Fig. 2) are consistent with our previous proposal [5] that there were three independent origins for *japonica*, *indica* and *aus*, with each of these cultivated groups acquiring the crucial domestication characteristics without a contribution from gene flow. Our results suggest that most domestication alleles were present in the wild progenitor populations of all three groups, and selection from standing variation was the major force in rice domestication. Signatures of gene flow are apparent in all three cultivated gene pools and several domestication-related loci were exchanged between the groups, particularly in case of *aus*. Nonetheless, it appears that the introgressions into the genomes of *indica* and *japonica* occurred at later stages of rice cultivation, perhaps quite recently, and that transcontinental cultural contacts – a prerequisite of such gene flow – were not necessary for the emergence of agriculture in different parts of Asia.

## Additional files

Additional file 1: Supporting tables. (**Tables S1** and **S2**). (XLS 190 kb)

Additional file 2: Supporting figures. **Figure S1.** Schematic summary of the data processing and analysis pipeline. **Figure S2.** Variants shared and fixed in cultivated groups. Alleles that are simultaneously fixed in *indica*, *japonica* and *aus* (counts shown on *y* axis) are always found in wild populations, usually with high allelic frequencies (*x* axis). **Figure S3.** Histogram of SNP densities. Histograms show the number of sites per 100 kb window used for the calculation of the introgression index shown on Fig. 4. Mean number of sites per window is shown for each dataset in corresponding colours. (PDF 119 kb)

## Abbreviations

AFS: Allele frequency spectrum
CLDGR: Co-located low diversity region
ML: Maximum likelihood
PCA: Principal component analysis
SNP: Single nucleotide polymorphism
