# Supplementary material for: Role of genetic introgression during the evolution of cultivated rice (Oryza sativa L.)
Source: BMC Evol Biol. 2018 Apr 23;18:57. doi: 10.1186/s12862-018-1180-7 (PMC5913815; doi:10.1186/s12862-018-1180-7)
Supplement: Supplementary file 2 — Supporting figures. Figure S1. Schematic summary of the data processing and analysis pipeline. Figure S2. Variants shared and fixed in cultivated groups. Alleles that are simultaneously fixed in indica, japonica and aus (counts shown on y axis) are always found in wild populations, usually with high allelic frequencies (x axis). Figure S3. Histogram of SNP densities. Histograms show the number of sites per 100 kb window used for the calculation of the introgression index shown on Fig. 4. Mean number of sites per window is shown for each dataset in corresponding colours. (PDF 119 kb) [file 12862_2018_1180_MOESM2_ESM.pdf]

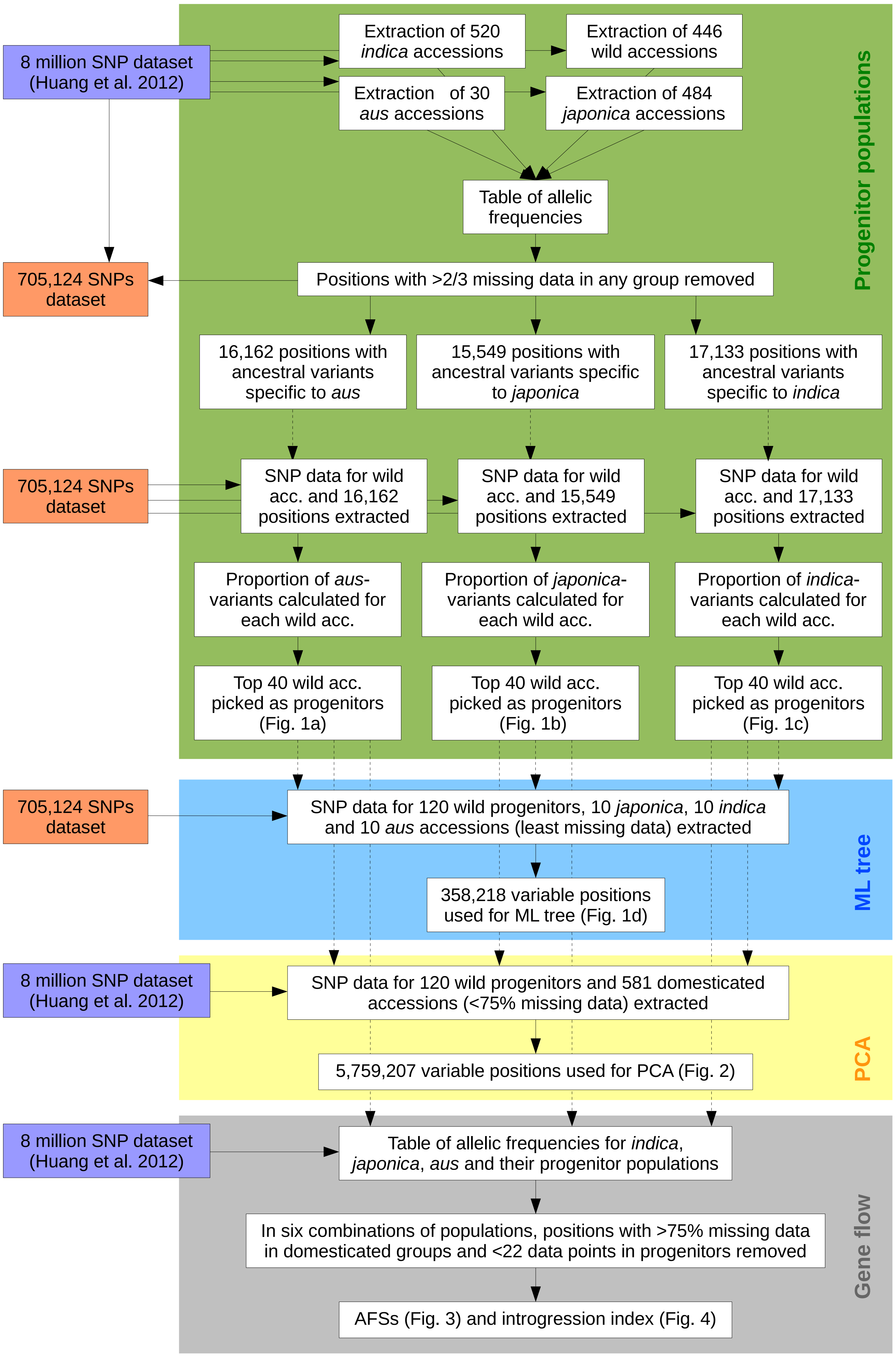

Figure S1: Schematic summary of the data processing and analysis pipeline.

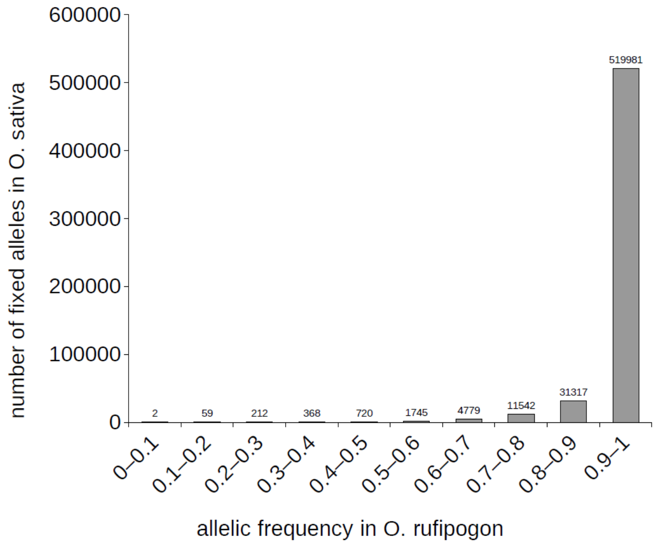

Figure S2: Variants shared and fixed in cultivated groups. Alleles that are simultaneously fixed in *indica*, *japonica* and *aus* (counts shown on *y* axis) are always found in wild populations, usually with high allelic frequencies (*x* axis).

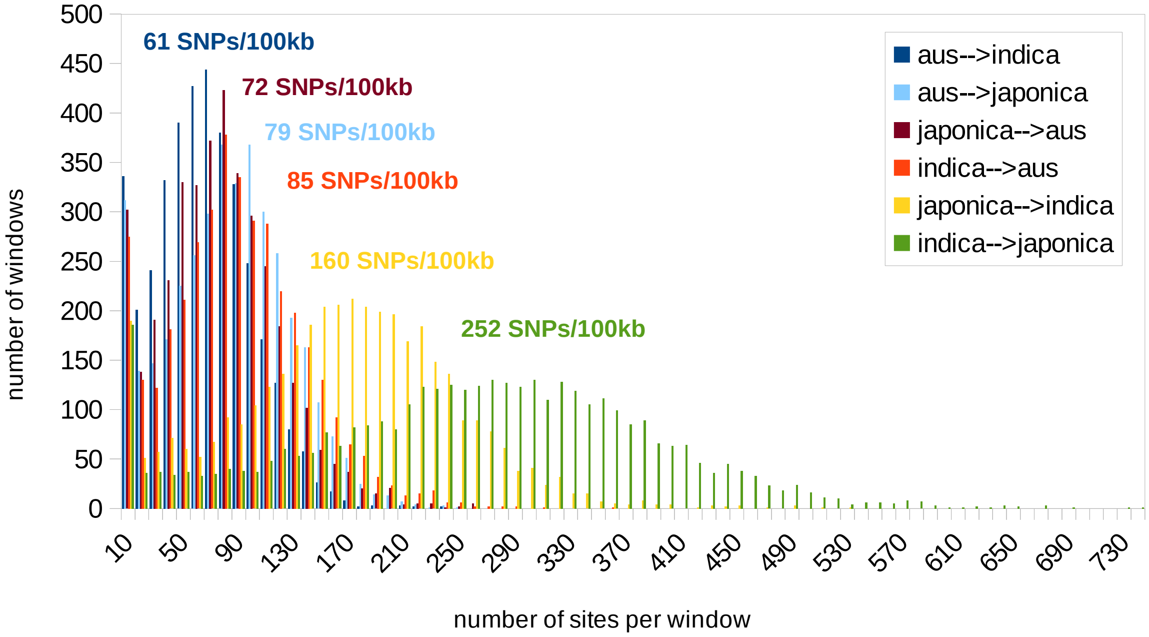

Figure S3: Histogram of SNP densities. Histograms show the number of sites per 100 kb window used for the calculation of the introgression index shown on Fig. 4. Mean number of sites per window is shown for each dataset in corresponding colours.
